# The Effects of Percutaneous Endoscopic Gastrostomy on Quality of Life in Patients With Dementia

**DOI:** 10.4021/gr392w

**Published:** 2012-01-20

**Authors:** Yutaka Suzuki, Mitsuyoshi Urashima, Masaki Izumi, Yasuhiko Ito, Nobuyuki Uchida, Shingo Okada, Hiromi Ono, Satoshi Orimo, Takayuki Kohri, Hiroaki Shigoka, Shuzo Shintani, Yukiko Tanaka, Atsushi Yoshida, Masashi Ijima, Toru Ito, Takao Endo, Hitoshi Okano, Michio Maruyama, Tsuyoshi Iwase, Tsutomu Kikuchi, Michiaki Kudo, Mikako Takahashi, Satoshi Goshi, Tatsuya Mikami, Satoyoshi Yamashita, Kazuhiro Akiyama, Tetsushi Ogawa, Tomoko Ogawa, Shigeki Ono, Shigeru Onozawa, Junya Kobayashi, Masami Matsumoto, Toshifumi Matsumoto, Kazuaki Jomoto, Akihiro Mizuhara, Yukio Nishiguchi, Shinji Nishiwaki, Masahiko Aoki, Izumi Ishizuka, Toshiroh Kura, Masato Murakami, Akihiko Murakami, Tomoyuki Ohta, Koji Onishi, Masato Nakahori, Tsuyotoshi Tsuji, Ko Tahara, Ikuta Tanaka, Kazuhiko Kitagawa, Makoto Shimazaki, Takanori Fujiki, Toshiro Kusakabe, Takao Iiri, Shuichirou Kitahara, Akira Horiuchi, Hitoshi Suenaga, Naohiro Washizawa, Masahiko Suzuki

**Affiliations:** 1 Department of Surgery, International University of Health and Welfare Hospital 537-3 Iguchi, Nasushiobara-shi, Tochigi 329-2763, Japan; 2 Department of Molecular Epidemiology, Jikei University School of Medicine, 3-25-8 Nishi-shimbashi, Minato-ku, Tokyo, 105-8461, Japan; 3 Department of Internal Medicine, Tokyo-Musashino Hospital, 4-11-11 Komone, Itabashi-ku, Tokyo, 173-0037, Japan; 4 Suzuki Internal Medicine Clinic, 4-5-43 Heiwadai, Nagareyama-shi, Chiba, 270-0157, Japan; 5 Department of Surgery, Haramachi Red-Cross Hospital, 698 Haramachi, Higashi-Agatsuma-machi, Gunma, 377-0882, Japan; 6 Department of Surgery, Kitamihara Clinic, 18-350 Ishikawa-cho, Hakodate-shi, Hokkaido, 041-0802, Japan; 7 Department of Internal Medicine, Seiwa Memorial Hospital, 5-1-1 Kotoni1,Nishi-ku, Sapporo-shi, Hokkaido, 063-0811, Japan; 8 Department of Neurology, Kanto Central Hospital, 6-25-1 Kami-Yoga, Setagaya-ku, Tokyo, 158-8531, Japan; 9 Department of Surgery, Tone chuou hospital, 1855-1 Higashiharashinmachi, Numata-shi, Gunma, 378-005, Japan; 10 Division of Gastroenterology, Department of Internal Medicine, Toho University Ohashi Medical Center, 2-17-6 Ohashi, Meguro-ku, Tokyo, 153-8515, Japan; 11 Department of Neurology, JA Toride Medical Center, 2-1-1 Hongoh, Toride City, Ibaraki, 302-0022, Japan; 12 The Internal Department, Uchida hospital, 345-1 Kuyahara, Numata-shi, Gunma, 378-0005, Japan; 13 Center for Digestive and Liver Diseases, Ohfuna Chuo Hospital, 6-2-24 Ohfuna, Kamakura, Kanagawa, 247-0056, Japan; 14 Department of Internal Medicine, Isesaki Municipal Hospital, 12-1 Tsunatorihonmachi, Isesaki-shi, Gunma, 372-0812, Japan; 15 Department of Surgery, Nakamura Memorial Hospital, Nishi-14,Minami-1,Chuo-ku, Sapporo-shi, Hokkaido, 060-8570, Japan; 16 Department of Gastroenterology, Sapporo Shirakaba-dai hospital, 2-18-7-26 Tukisamu-Higash,Toyohira-ku, Sapporo-shi, Hokkaido, 062-0052, Japan; 17 Department of Gastroenterology, Okano clinic, 3 Higashida-cho,Jodoji, Sakyo-ku, Kyoto-shi, Kyoto, 606-8411, Japan; 18 Deaprtment of Surgery, Tokyo Metropolitan Ohkubo Hospital, 2-44-1 Kabukicho, Sinnjyukuku, Tokyo, 160-8488, Japan; 19 Department of Internal medicine, Kyoto kujo hospial, 10 Karahashi Rajomon-cho Minami-ku, Kyoto-shi, Kyoto, 601-8485, Japan; 20 Department of Surgery, Kanazawa Nishi Hospital, 6-15-41 Ekinishihonmachi, Kanazawa-shi, Ishikawa, 920-0025, Japan; 21 Department of Surgery, Onishi Hospital, 139-1 Onishi, Fujika-shi, Gunma, 370-1401, Japan; 22 Department of Internal Medicine, Tsuruika Kyouritsu Hospotal, 9-34 Fumizono-cho, Tsuruoka-shi, Yamagata, 997-0816, Japan; 23 Department of Gastroenterology and Hepatology, Japan Agricultural Cooperatives of Niigata Joetsu General Hospital, 148-1 Daidoufukuda, Joetsu-shi, Niigata, 943-8507, Japan; 24 Department of Gastroenterology, Hirosaki Municipal Hospital, 1-8-3 Ohmachi, Hirosaki- shi, Aomori, 036-8004, Japan; 25 Department of Gastroenterology, Social Insurance Shimonoseki Welfare Hospital, 3-3-8 Kamishinchi, Shimonoseki-shi, Yamaguchi, 750-0061, Japan; 26 Department of Surgery, Tokatsu-clinic Hospital, 865-2 Hinokuchi, Matsudo-shi, Chiba, 271-0067, Japan; 27 Department of Surgery, Maebashi Red Cross Hospital, 3-21-36 Asahi-cho, Maebashi-shi, Gunma, 371-0014, Japan; 28 Department of Neurology, International University of Health and Welfare Hospital, 537-3 Iguchi, Nasushiobara-shi, Tochigi, 329-2763, Japan; 29 Department of Gastroenterology, Ako City Hospital, 1090 Nakahiro, Ako-shi, Hyogo, 678-0232, Japan; 30 Department of HomeCareMedicine, Kameda Medical Center, 929 Higashicho, Kamogawa-shi, Chiba, 292-8601, Japan; 31 Department of Surgery, Fujiyoshida Municipal Hospital, 6530 Kamiyoshida, Fujiyoshida-shi, Yamanashi, 403-0005, Japan; 32 Department of Internal Medicine, Nara Prefectural Gojo Hospital, 5-2-59 Noharanishi, Gojo-shi, Nara, 637-8511, Japan; 33 Department of Surgery, National Hospital Organization Beppu Medical Center, 1473 Uchikamado, Beppu, Oita, 874-0011, Japan; 34 Director, Jomoto Gastroenteric & Internal Medical Clinic, 5-8-9 Kuhonnji, Kumamoto-shi, Kumamoto, 862-0976, Japan; 35 Department of Cardiology and Vascular Surgery, Higashi-Washinomiya Hospital, 3-9-3 Sakurada, Kuki-shi, Saitama, 340-0203, Japan; 36 Department of Gastro-enterological Surgery, Osaka City General Hospital, 2-13-22 Miyakojima-Hondori Miyakojima-ku, Osaka-shi, Osaka, 534-0021, Japan; 37 Department of Internal Medicine, Nishimino Kosei Hospital, 986 Oshikoshi, Yoro-gun Yoro-cho, Gifu, 503-1394, Japan; 38 Department of Surgery, Ohtawara Red Cross Hospital, 2-7-3 Sumiyoshi-cho, Ohtawara-shi, Tochigi, 324-8686, Japan; 39 Department of Internal medicine, Kohka Public Hospital, 3-39 Rokushin,Minakuchi-cho, Kohka-shi, Shiga, 528-0014, Japan; 40 Department of Gastroenterology, Naganuma Municipal Hospital, 2-2-1 Chuo-minami,Naganuma-cho, Yubari-gun, Hokkaido, 069-1332, Japan; 41 Department of Internal Medicine, Murakami Memorial Hospital, 739 Oomachi, Saijo-shi, Ehime, 793-0030, Japan; 42 Endoscopy unit, Iwate prefectural central Hospital, 1-4-1 ueda, Morioka-shi, Iwate, 020-0066, Japan; 43 Center for Gastroenterology, Sapporo Higashi-Tokushukai Hospital, 3-1 North33-East14,Higashi-ku, Sapporo-shi, Hokkaido, 065-0033, Japan; 44 Internal Medicine, Matsue Seikyo General Hospital, 8-8-8 Nishitsuda, Matsue-shi, Shimane, 690-8522, Japan; 45 Digestive Endoscopy Center, Sendai Kousei Hospital, 4-8 Hirose-machi,Aoba-ku, Sendai-shi, Miyagi, 980-0873, Japan; 46 Department of Gastroenterology, Akita City Hospital, 4-30 Kawamotomatsuoka-cho, Akita-shi, Akita, 010-0933, Japan; 47 Department of Surgery, Kure Kyosai Hospital, 2-3-28 Nishichuou, Kure-shi, Hiroshima, 737-8505, Japan; 48 Department of Gastroenterology, Japan Red Cross Date General Hospital, 81 Suenaga, Date-shi, Hokkaido, 052-8511, Japan; 49 Department of Surgery, Tsuchida Hospital, 2-11,South-21,West-9,Chuo-ku, Sapporo-shi, Hokkaido, 064-0921, Japan; 50 Department of Gastroenterology, Hirano General Hospital, 176-5 Kurono, Gifu-shi, Gifu, 501-1192, Japan; 51 Department of Gastroenterology, Japanese Red Cross Society Shimizu Hospital, 2-2 minami,Shimizu-cho, Kamikawa-gun, Hokkaido, 089-0195, Japan; 52 Department of Gastroenterology, Higashi Sapporo Hospital, 3-3-7-35 Higashi-Sapporo,Shiroishi-ku, Sapporo-shi, Hokkaido, 003-8585, Japan; 53 Department of Gastroenterology, Tachikawa General Hospital, 3-2-11 Kandamachi, Nagaoka-shi, Niigata, 940-8621, Japan; 54 Department of Pediatric Surgery, Nagano Red Cross Hospital, 5-22-1 Wakasato, Nagano-shi, Nagano, 380-8582, Japan; 55 Digestive Disease Center, Showa Inan General Hospital, 3230 Akaho, Komagane-shi, Nagano, 399-4117, Japan; 56 Department of Surgery, Hitachikoh Hospital, 3-4-22 Kujichou, Hitachi-shi, Ibaraki, 319-1222, Japan; 57 Nutritional Therapy Center, Toho University Omori Medical Center, 6-11-1 Omorinishi, Ota-ku, Tokyo, 143-8541, Japan; 58 Department of Neurology, The Jikei University School of Medicine, Aoto Hospital, 6-41-2 Aoto, Katsushika-ku, Tokyo, 125-8506, Japan

**Keywords:** Alzheimer’s dementia, Cerebrovascular dementia, Percutaneous endoscopic gastrostomy, Quality of life, Risk factor

## Abstract

**Background:**

To examine the effects of percutaneous endoscopic gastrostomy (PEG) on quality of life (QOL) in patients with dementia.

**Methods:**

We retrospectively included 53 Japanese community and tertiary hospitals to investigate the relationship between the newly developed PEG and consecutive dementia patients with swallowing difficulty between Jan 1st 2006 and Dec 31st 2008. We set improvements in 1) the level of independent living, 2) pneumonia, 3) peroral intake as outcome measures of QOL and explored the factors associated with these improvements.

**Results:**

Till October 31st 2010, 1,353 patients with Alzheimer’s dementia (33.1%), vascular dementia (61.7%), dementia with Lewy body disease (2.0%), Pick disease (0.6%) and others were followed-up for a median of 847 days (mean 805 ± 542 days). A total of 509 deaths were observed (mortality 59%) in full-followed patients. After multivariate adjustments, improvement in the level of independent living was observed in milder dementia, or those who can live independently with someone, compared with advanced dementia, characterized by those who need care by someone: Odds Ratio (OR), 3.90, 95% confidence interval (95%CI), 1.59 - 9.39, P = 0.003. Similarly, improvement of peroral intake was noticed in milder dementia: OR, 2.69, 95%CI, 1.17 - 6.17, P = 0.02. Such significant associations were not observed in improvement of pneumonia.

**Conclusions:**

These results suggest that improvement of QOL after PEG insertion may be expected more in milder dementia than in advanced dementia.

## Introduction

Percutaneous endoscopic gastrostomy (PEG) was initially developed as one of enteral nutrition techniques for patients with swallowing difficulty, because of reduced laryngopharyngeal discomfort and a lower risk of aspiration pneumonia compared with the nasogastric tube. Randomized studies in patients after stroke who received gastrostomy feeding have shown improved nutritional outcomes, higher likelihood of survival, and earlier discharge [1, 2]. Nowadays, numbers of PEG placement as well as its replacement are rapidly growing in Japanese aging society. In our previous study [3], using Cox proportional multi-variate analysis, we determined the risk and beneficial factors for death among Japanese geriatric patients with PEG including not only dementia but also other conditions in long term follow-up: Older ages, higher CRP and higher BUN were significant poor prognostic factors of death after PEG formation, whereas higher albumin, female gender and no previous history of ischemic heart disease were obviously better prognostic factors.

Although patients with advanced-dementia commonly develop feeding problems that lead to weight loss and nutritional deficiencies, they lack the capacity to express their wishes, thus leaving the decision of PEG usage up to family members and doctors. A Cochrane review showed no evidence of increased survival or improved quality of life (QOL) in patients with advanced dementia who were fed using gastrostomy tubes [4]. On the other hand, in a letter to the editor [5], Leeds et al implied that PEG in patients with early dementia (i.e., outpatient) may be beneficial compared to PEG in patients that have more advanced disease (i.e., inpatient). In our previous work [3], we surveyed geriatric patients with PEG and focused on long term survivals. However, in this study, we surveyed patients with dementia and focused on improvement of QOL and explored the factors associated with these improvements.

## Patients and Methods

### Study design and population

We conducted a retrospective cohort study of patients who underwent PEG between Jan 1st 2006 and Dec 31st 2008 at 46 community hospitals all over Japan, selected by a panel of 104 doctor-experts in PEG and the trustees of PEG Doctors’ Network, a non-profit organization, which was approved by the institutional review board in each hospital. Doctors in charge of PEG in the selected hospitals were asked to examine patients with dementia with a new PEG, after excluding patients 1) who had gastrectomy in previous history, 2) who had cancer considered to affect the patient’s prognosis, 3) who were performed the gastrostomy without reason of nutrition-support. The doctors were further asked to report the number of excluded cases as well as the number of patients who were considered as loss to follow-up.

### Outcome measure

The primary endpoint was the improvement in 1) the level of independent living, 2) pneumonia, 3) peroral intake as outcome measures of QOL and the factors associated with these improvements were also explored. The level of independent living of the demented elderly was defined by Japanese Ministry of Health, Labor and Welfare [6, 7] (Table 1).

**Table 1:** The Level of Independent Living of Demented Elderly

I	Daily living is almost independent in family and community
II	There are some difficulties in daily life due to dementia-specific signs, behavior and communication, but the person can live independently with someone.
IIa	The difficulties are observed outside of home
IIb	The difficulties are observed inside of home
III	Difficulties in daily life due to dementia-specific signs, behavior and communication are sometimes observed, and the person needs care by someone.
IIIa	The difficulties are observed outside of home
IIIb	The difficulties are observed inside of home
IV	Difficulties in daily life due to dementia-specific signs, behavior and communication are frequently observed, and the person always needs care by someone.
M	Due to serious mental illness and problematic behavior or diseases, the person needs medical care by specialists.

The secondary outcome was set as death and the cutoff date was set at October 2010. In the case where the patient was alive, the doctor was further asked the status of the patient which was selected from the following: a) admission in the current hospital, b) admission in another hospital, c) stay at nursery home, d) stay at home, e) others. In case of loss to follow-up, the final date the patient was confirmed as alive was set a censor.

### Variables

Following data: 1) age, 2) gender, 3) height, 4) weight, 5) body temperature, 6) white blood cell (WBC/uL), 7) hematocrit (Ht): %, 8) hemoglobin (Hg): g/dL, 9) aspartate aminotransferase (AST): IU/L, 10) alanine aminotransferase (ALT): IU/L, 11) blood urine nitrogen (BUN): mg/dL, 12) creatinin (Cr): mg/dL, 13) albumin: g/dL, 14) C-reactive protein (CRP): mg/dL, 15) previous history of pneumonia, ischemic heart disease, 16) comorbidity of diabetes, serious malnutrition judged by the doctor in charge, 17) starvation period before making PEG: none, within 1 week, within 1 month, more than 1 month, 18) primary type of dementia executable for PEG, selected from the following: a) Alzheimer’s dementia, b) cerebrovascular dementia, c) Dementia with Lewy bodies, d) Pick disease, e ) other type of dementia.

### Statistics analysis

Student’s *t*-test and Mann-Whitney test were performed for continuous variables with normal and not normal distribution, respectively. Chi-square test was calculated for dichotomous outcomes. Primary outcome was evaluated with multiple logistic regression models with multi-variate adjustments using odds ratio (OR) and 95% confidence intervals (95%CI). Cox proportional hazard models were fitted for either single or multivariate analysis using variables significant at single analyses. All statistical analyses were performed using STATA 11.0 (STATA Corp., College Station, TX). P < 0.05 was considered statistically significant.

## Results

Of the 1,353 patients who underwent PEG at the selected 53 hospitals. Their mean age was 81.9 years old, which ranged from 23 to 104. Females were predominant (60%). The distribution of primary diagnosis executable for PEG was as follows: Cerebrovascular dementia, 62%: Alzheimer’s dementia, 33%, dementia with Lewy bodies, 2%. In the previous history, pneumonia and ischemic heart disease were reported at 68% and 16%, respectively. Moreover, comorbidity of diabetes, hypertension, dyslipidemia, was 16%, 41%, and 10%. In 865 patients who were able to be fully followed-up, 509 deaths were observed (mortality 59%). Among 1027 patients including cases censored by moving to other hospitals, 99%, 95%, 90%, 75% and 50% survived more than 11 days, 32 days, 65 days, 268 days and 847 days, respectively.

In total, 509 deaths were observed, of which 8 deaths (1.6% of total death) occurred within 7 days, 50 (9.8%) within 30 days, 99 (19%) within 60 days, 207 (41%) within a half year, and 305 (60%) within one year. Seven deaths were considered as PEG related deaths, according to the reported doctors. On the other hand, among 28 surviving patients (6.5%), PEG was removed. Among the 1,353 patients, 879, 961 and 777 patients were evaluated for improvement of the level of independent living of the demented elderly, peroral intake, and pneumonia, respectively.

First, variables of demographic and laboratory data at PEG installation were compared between improved and not improved patients in the level of independent living of the demented elderly (Table 2). Only Age was the significant factor and younger patients had improved levels as opposed to older patients. Improvement of the level of independent living was assessed by stratification with the level of independent living at the beginning of PEG (Table 3). Dementia stages IIa and IIb showed a significantly higher ratio (OR = 3.9, 95%CI, 1.6 to 9.4, P = 0.003) compared with dementia stages IIIa and IIIb, even after adjustment with age, which was significantly associated with improvement of the level of independent living.

**Table 2:** Patients’ Characteristics at Making PEG Stratified by the Level of Independent Living of Demented Elderly

Variable	Total (N = 879)	Improved (N = 75)	Not improved (N = 804)	P-value
Age (years) mean ± s.d.	81.8 ± 9.4	79.5 ± 13.0	82.0 ± 8.9	0.02^*1^
Body Temperature (°C) mean ± s.d.	36.8 ± 0.6	36.8 ± 0.5	36.8 ± 0.6	0.68^*1^
WBC ( /uL)	7026 ± 2567	7354 ± 2593	6995 ± 2564	0.12^*2^
CRP (mg/dL)	1.86 ± 2.40	1.81 ± 2.10	1.87 ± 2.42	0.73^*2^
Hb (g/dL)	11.1 ± 1.9	11.2 ± 1.9	11.1 ± 1.9	0.49^*2^
ALT (IU/L)	29.8 ± 26.5	25.8 ± 12.5	30.2 ± 27.4	0.46^*2^
BUN (mg/dL)	21.6 ± 13.7	20.7 ± 12.2	21.8 ± 14.1	0.60^*2^
Cr (mg/dL)	0.84 ± 1.15	0.80 ± 0.86	0.85 ± 1.21	0.05^*2^
Albumin (g/dL)	3.01 ± 0.57	3.03 ± 0.58	3.01 ± 0.55	0.88^*2^
Total cholesterol (mg/dL)	157.2 ± 40.8	162.3 ± 38.6	156.0 ± 41.2	0.58^*2^
male	359 (41%)	32 (43%)	327 (40%)	0.70^*3^
Previous history of pneumonia	587 (68%)	50 (68%)	537 (68%)	0.94^*3^
Hypertension	348 (40%)	35 (47%)	313 (39%)	0.18^*3^
Dyslipidemia	77 (9%)	9 (13%)	68 (9%)	0.28^*3^
Arteriosclerosis obliterans	31 (3.7%)	5 (6.9%)	26 (3.4%)	0.14^*3^
Able to take perorally	324 (37%)	31 (41%)	293 (37%)	0.44^*3^
Diabetes	124 (14%)	11 (15%)	113 (14%)	0.90^*3^
Previous history of cardiovascular disease	134 (16%)	12 (16%)	122 (16%)	0.91^*3^
Extremely poor nutritional status	170 (20%)	16 (22%)	154 (19%)	0.66^*3^
Fasting period prior to operation				0.72^*3^
None	189 (28%)	12 (25%)	177 (29%)	
Within one week	292 (44%)	24 (50%)	268 (43%)	
Within one month	177 (27%)	12 (25%)	165 (27%)	
More than one month	8 (1.2%)	0 (0%)	8 (1.3%)	
Alzheimer’s dementia	292 (33%)	27 (36%)	265 (33%)	0.56^*3^
Cerebrovascular dementia	554 (63%)	43 (57%)	511 (63%)	0.33^*3^
Dementia with Lewy bodies	15 (1.7%)	1 (1.3%)	14 (1.7%)	0.80^*3^

*1. Student’s t-test was applied because the distribution was considered as normal. *2. Mann-Whitney U test was applied because the distribution was considered as not normal. *3. Chi-square test was applied.

**Table 3:** Improvement of the Level of Independent Living Stratified by the Level of Independent Living at Making PEG

Variable	I	IIa/IIb	IIIa/IIIb/IV	M
No. of improved patients/no. of total patients (%)	0/2 (0)	8/32 (25)	32/375 (8.6)	7/57 (12)
Odds Ratio (95% confidence interval)*	-	3.90	1	1.36
P-value		(1.59 - 9.39)		(0.56 - 3.30)
		0.003		0.50

*Odds Ratio was adjusted with age, which was associated with the outcome.

Next, variables of demographic and laboratory data at PEG installation were compared between improved and not improved patients in ability of peroral intake (Table 4). Patients with dyslipidemia and ability of peroral intake tended to have improved in the level of independent living. Patients with Alzheimer’s dementia improved significantly more than those with cerebrovascular dementia. Improvement of the peroral intake was assessed by stratification with the level of independent living at the beginning of PEG (Table 5). Dementia stages IIa and IIb showed a significantly higher ratio (OR = 2.7, 95%CI, 1.1 to 6.2, P = 0.02) as compared with dementia stages IIIa and IIIb, even after adjustment with dyslipidemia, peroral intake, Alzheimer’s dementia and cerebrovascular dementia that were significantly associated with improvement of the level of independent living.

**Table 4:** Patients’ Characteristics at Making PEG Stratified by the Improvement of Peroral Intake

Variable	Total (N = 961)	Improved (N = 177)	Not improved (N = 784)	P-value
(Age years) mean ± s.d.	81.7 ± 9.3	80.6 ± 11.1	82.0 ± 8.8	0.07^*1^
Body Temperature (°C) mean ± s.d.	36.8 ± 0.6	36.8 ± 0.6	36.8 ± 0.6	0.12^*1^
WBC ( /µL)	7041 ± 2702	6869 ± 2364	7080 ± 2773	0.62^*2^
CRP (mg/dL)	1.90 ± 2.38	1.69 ± 2.08	1.95 ± 2.45	0.39^*2^
Hb (g/dL)	11.6 ± 1.9	11.1 ± 2.1	11.1 ± 1.9	0.99^*2^
ALT (IU/L)	30.4 ± 28.7	29.6 ± 34.7	30.6 ± 27.2	0.13^*2^
BUN (mg/dL)	21.5 ± 13.8	21.3 ± 14.9	21.5 ± 13.7	0.94^*2^
Cr (mg/dL)	0.83 ± 1.16	0.70 ± 0.36	0.84 ± 1.20	0.09^*2^
Albumin (g/dL)	3.02 ± 0.57	3.03 ± 0.46	3.02 ± 0.58	0.52^*2^
Total cholesterol (mg/dL)	157.5 ± 40.6	160.7 ± 37.1	157.2 ± 40.8	0.14^*2^
male	396 (41%)	66 (37%)	330 (42%)	0.24^*3^
Previous history of pneumonia	645 (68%)	107 (62%)	538 (70%)	0.051^*3^
Hypertension	389 (41%)	78 (45%)	311 (40%)	0.29^*3^
Dyslipidemia	88 (10%)	30 (17%)	58 (8%)	< 0.001^*3^
Arteriosclerosis obliterans	31 (3.4%)	8 (4.7%)	23 (3.1%)	0.29^*3^
Able to take perorally	350 (37%)	95 (54%)	255 (33%)	< 0.001^*3^
Diabetes	150 (16%)	35 (20%)	115 (15%)	0.093^*3^
Previous history of cardiovascular disease	152 (16%)	29 (17%)	123 (16%)	0.80^*3^
Extremely poor nutritional status	189 (20%)	39 (22%)	147 (19%)	0.34^*3^
Fasting period prior to operation				0.11^*3^
None	216 (30%)	46 (34%)	170 (29%)	
Within one week	316 (43%)	65 (47%)	251 (42%)	
Within one month	188 (26%)	25 (18%)	163 (27%)	
More than one month	12 (1.6%)	1 (0.7%)	11 (1.9%)	
Alzheimer’s dementia	310 (32%)	76 (43%)	234 (30%)	0.001^*3^
Cerebrovascular dementia	610 (63%)	95 (53%)	515 (65%)	0.003^*3^
Dementia with Lewy bodies	18 (1.9%)	2 (1.1%)	16 (2.0%)	0.42^*3^

*1. Student’s t-test was applied because the distribution was considered as normal. *2. Mann-Whitney U test was applied because the distribution was considered as not normal. *3. Chi-square test was applied.

**Table 5:** Improvement of Peroral Intake Stratified by the Level of Independent Living at Making PEG

Variable	I	IIa/IIb	IIIa/IIIb/IV	M
No. of improved patients/no. of total patients (%)	1/2 (50)	12/34(35)	70/419 (17)	13/62 (21)
Odds Ratio (95% confidence interval)*	-	2.69	1	1.39
P-value		(1.17 – 6.17)		(0.70 -2.77)
		0.02		0.35

*Odds Ratio was adjusted with dyslipidemia, able to take perorally, Alzheimer’s dementia, and cerebrovascular dementia, which were associated with the outcome.

Then, variables of demographic and laboratory data at PEG installation were compared between improved and not improved patients with pneumonia (Table 6). Patients who have previous history of pneumonia or cardiovascular disease as well as dyslipidemia and/or diabetes had more improvement in pneumonia. Improvement of pneumonia was assessed by stratification with the level of independent living at PEG installation (Table 7). However, improvement of pneumonia in dementia with IIa and IIb was not significantly different from that in dementia with IIIa and IIIb, even after adjustment with dyslipidemia, peroral intake, Alzheimer’s dementia and cerebrovascular dementia which were factors significantly associated with improvement of the level of independent living.

**Table 6:** Patients’ Characteristics at Making PEG Stratified by the Improvement of Pneumonia

Variable	Total (N = 777)	Improved (N = 557)	Not improved (N = 220)	P-value
Age (years) mean ± s.d.	82.1 ± 9.0	82.1 ± 9.3	81.9 ± 8.4	0.73^*1^
Body Temperature (°C) mean ± s.d.	36.8 ± 0.6	36.8 ± 0.6	36.8 ± 0.6	0.79^*1^
WBC (/µL)	7101 ± 2745	7222 ± 2847	6797 ± 2454	0.08^*2^
CRP (mg/dL)	2.01 ± 2.45	2.00 ± 2.43	2.05 ± 2.04	0.85^*2^
Hb (g/dL)	11.0 ± 1.9	11.0 ± 1.8	10.9 ± 2.0	0.47^*2^
ALT(IU/L)	30.7 ± 29.5	30.6 ± 30.7	30.7 ± 26.4	0.60^*2^
BUN (mg/dL)	21.5 ± 13.4	21.3 ± 12.7	21.8 ± 14.9	0.63^*2^
Cr (mg/dL)	0.84 ± 1.15	0.85 ± 1.17	0.83 ± 1.09	0.92^*2^
Albumin (g/dL)	2.98 ± 0.58	2.97 ± 0.54	3.01 ± 0.68	0.69^*2^
Total cholesterol (mg/dL)	155.5 ± 40.3	154.5 ± 40.1	158.1 ± 40.8	0.29^*2^
male	343 (44%)	240 (43%)	103 (46%)	0.38^*3^
Previous history of pneumonia	628 (81%)	480 (86%)	148 (69%)	<0.001^*3^
Hypertension	315 (41%)	225 (41%)	90 (41%)	0.99^*3^
Dyslipidemia	73 (10%)	61 (11%)	12 (6%)	0.015^*3^
Arteriosclerosis obliterans	28 (3.8%)	19 (3.6%)	9 (4.2%)	0.70^*3^
Able to take perorally	270 (35%)	188 (34%)	82 (38%)	0.29^*3^
Diabetes	113 (15%)	93 (17%)	20 (9%)	0.006^*3^
Previous history of cardiovascular disease	121 (16%)	98 (18%)	23 (11%)	0.01^*3^
Extremely poor nutritional status	158 (21%)	106 (20%)	52 (24%)	0.20^*3^
Fasting period prior to operation				
None	170 (29%)	130 (32%)	40 (24%)	
Within one week	244 (42%)	147 (36%	97 (57%)	
Within one month	159 (27%)	130 (32%)	29 (17%)	
More than one month				
Alzheimer’s dementia	248 (32%)	182 (33%)	66 (30%)	0.45^*3^
Cerebrovascular dementia	484 (62%)	340 (61%)	144 (65%)	0.28^*3^
Dementia with Lewy bodies	13 (1.7%)	10 (1.8%)	3 (1.4%)	0.67^*3^

*1. Student’s t-test was applied because the distribution was considered as normal. *2. Mann-Whitney U test was applied because the distribution was considered as not normal. *3. Chi-square test was applied.

**Table 7:** Improvement of Pneumonia Stratified by the Level of Independent Living at Making PEG

Variable	I	IIa/IIb	IIIa/IIIb/IV	M
No. of improved patients/no. of total patients (%)	1/1 (100)	18/27 (67)	229/351 (65)	30/50 (60)
Odds Ratio (95% confidence interval)*	-	1.33	1	0.61
P-value		(0.45 - 3.89)		(0.28 - 1.31)
		0.60		0.20

*Odds Ratio was adjusted with previous history of pneumonia, dyslipidemia, diabetes, previous history of cardiovascular disease and fasting period prior to operations, which were associated with the outcome.

Finally, using variables significant in above analyses, Cox proportional hazard models were computed in single- and multi-variate analyses (Table 8). In single variate hazard models, patients with older ages, male patients, higher CRP, AST and BUN did show a significantly enhanced crude hazard ratio, whereas higher Hb, albumin, and total cholesterol reduced the crude hazard ratio. Moreover, history of pneumonia or ischemic heart disease and extremely poor nutritional status increased, but ability of peroral intake significantly decreased crude hazard ratios of death. However, in a multivariate hazard model using significant factors in single variate analyses, older ages, higher BUN, lower albumin, male gender and diabetes were significant risk factors of death after PEG formation.

**Table 8:** Cox Proportional Hazard Models

Variable	Single-variate analyses			Multivariate analysis		
	Crude HR	95% CI	P value	AHR	95% CI	P value
Age (year)	1.03	1.02 - 1.04	< 0.001	1.04	1.02 - 1.06	< 0.001
Body mass index (kg/m^2^)	0.96	0.93 - 0.99	0.04	0.98	0.94 - 1.03	0.46
Body Temperature (°C)	0.93	0.80 - 1.09	0.39			
WBC (/uL)	1.00	0.99 - 1.00	0.31			
CRP (mg/dL)	1.07	1.03 - 1.10	< 0.001	1.00	0.95 - 1.06	0.86
Hb (g/dL)	0.88	0.84 - 0.92	< 0.001	1.03	0.94 - 1.14	0.48
ALT (IU/L)	1.00	1.00 - 1.01	0.001	1.00	0.99 - 1.01	0.10
BUN (mg/dL)	1.02	1.01 - 1.02	< 0.001	1.02	1.01 - 1.03	< 0.001
Cr (mg/dL)	1.04	0.98 - 1.11	0.23	0.94	0.84 - 1.06	0.30
Albumin (g/dL)	0.64	0.55 - 0.76	< 0.001	0.51	0.36 - 0.73	< 0.001
Total cholesterol (mg/dL)	0.99	0.99 - 1.00	0.001	1.00	0.99 - 1.00	0.90
Male	1.56	1.31 - 1.86	< 0.001	2.01	1.49 - 2.92	< 0.001
Previous history of pneumonia	1.42	1.16 - 1.73	0.001	1.10	0.79 - 1.54	0.56
level of independent living of demented elderly	1.05	0.97 - 1.15	0.25			
Hypertension	1.17	0.98 - 1.39	0.09			
Dyslipidemia	1.20	0.91 - 1.60	0.20			
Arteriosclerosis obliterans	1.21	0.78 - 1.88	0.39			
Able to take perorally	0.76	0.63 - 0.92	0.004	0.82	0.59 - 1.12	0.21
Diabetes	1.61	1.29 - 1.99	0.10	2.17	1.46 - 3.25	< 0.001
Previous history of cardiovascular disease	1.45	1.16 - 1.81	0.001	1.09	0.73 - 1.65	0.67
Extremely poor nutritional status	1.40	1.13 - 1.73	0.002	1.31	0.92 - 1.88	0.14
Fasting period prior to operation	1.05	0.98 - 1.11	0.14			
Alzheimer’s dementia	0.93	0.78 - 1.12	0.47			
Cerebrovascular dementia	1.17	0.98 - 1.41	0.08			
Dementia with Lewy bodies	0.81	0.40 - 1.63	0.55			
Level of independent living						
I	-	-				
IIa/IIb	0.59	0.34 - 1.00				
IIIa/IIIb	-	-				
M	1.03	0.72 - 1.47				

*Adjusted for all the variables listed in the table. HR, hazard ratio; CI, confidence interval; AHR, adjusted hazard ratio.

## Discussion

In this study, improvement in the level of independent living was observed in 8.5% of the whole and in 25% of milder dementia, or those who can live independently with someone, compared with 8.6% of advanced dementia, or those who need care by someone. The difference was almost 4 times between mild and advanced dementia, even after multivariate adjustment. Similarly, improvement of peroral intake was noticed in 18.4% of the whole and in 35% of the milder dementia, compared with 17% of advanced dementia. The difference was 2.7 times between them. Thus, severity of dementia may be a very important factor in the decision making of PEG formation. Improvement of pneumonia was observed in 72% of all patients who inserted PEG, but such significant associations with the level of dementia were not observed in the improvement of pneumonia. Yokoyama et al showed that a total of 15% of PEG cases were able to ingest orally after PEG [8], of which ratio was close to our data of 18%.

For survival analysis, more than half of dementia patients treated with PEG may survive more than 2.3 years, which is almost equivalent to our previous study [3] and better than a previous retrospective study of 361 patients which found that patients with dementia who had a PEG inserted had higher mortality than other patient subgroups (54% 30 day mortality and 90% at one year) [9]. In a multi-variate Cox hazard model, older age, male gender, comorbidity of diabetes, higher BUN and lower albumin were significant risk factors of death, which is not inconsistent with our previous results [3] and others [10, 11]. Of interest, important factors to predict improvement of QOL after PEG insertion such as levels of dementia and underlying disease of dementia were not included in these prognostic factors.

When the dementia levels are mild for patients enough to live independently with someone, insertion of PEG may improve the level of independent living and ability of peroral intake. However, these improvements were observed in some cases of advanced dementia. Moreover, prognostic factors for survival were different from key factors in improvement of QOL. In addition to these evidences, moral and ethical issues, as well as respecting the patient’s wishes should be considered in the decision making of PEG insertion. As Kurien et al [12] insisted, guidelines exist to aid clinicians in making decisions on PEG feeding, but the decision to insert a PEG tube should always be made on an individual basis.

The results of this study should be interpreted in the context of the study strengths and limitations. We researched in multiple community and tertiary hospitals spread over Japan, which enhanced generalizability. To minimize selection bias, collaborating doctors were asked to choose 20 consecutive patients. The sample size was 1,353 and the results of the statistical analyses were considered relatively robust. On the other hand, due to the retrospective nature of the study, we could collect only basic clinical information that might include recall bias in areas such as previous histories and diagnosis of underlying diseases. Most importantly, because this study is not randomized and just a single arm of PEG insertion, we can not conclude the superiority of PEG to nasogastric tube and peroral feeding.

In conclusion, these results suggest the following in QOL of patients: the level of independent living and peroral intake improved in patients with milder dementia, compared with patients with advanced dementia.
